# Epidemiological trends in respiratory pathogens infections among children post-COVID-19: A cross-sectional study

**DOI:** 10.1016/j.heliyon.2024.e36809

**Published:** 2024-08-23

**Authors:** Tiewei Li, Xiaojuan Li, Xinrui Liu, Lili Dong, Hui Fu, Fatao Lin, Yu Tang, Zhipeng Jin

**Affiliations:** a Department of Clinical Laboratory, Children's Hospital Affiliated to Zhengzhou University, Zhengzhou Key Laboratory of Children's Infection and Immunity, Zhengzhou, China; b Department of Respiratory Medicine, Children's Hospital Affiliated to Zhengzhou University, Henan Children's Hospital, Zhengzhou Children's Hospital, Zhengzhou, China; c Department of Neonatal Medicine, Children's Hospital Affiliated to Zhengzhou University, Henan Children's Hospital, Zhengzhou Children's Hospital, Zhengzhou, China; d Pediatric Intensive Care Unit, Children's Hospital Affiliated to Zhengzhou University, Henan Children's Hospital, Zhengzhou Children's Hospital, Zhengzhou, China

**Keywords:** Epidemiology, Influenza virus, Respiratory syncytial virus, Adenovirus, Mycoplasma pneumonia, Children, Post-COVID-19

## Abstract

**Objective:**

The aim of this study was to investigate the epidemiological trend of respiratory pathogens infections among children after the Coronavirus Disease 2019 (COVID-19) pandemic.

**Methods:**

This study enrolled 575,373 children who came to our hospital for relevant respiratory pathogen antigen/antibody testing due to respiratory symptoms such as fever and cough. The demographic and laboratory data, including age, gender, testing time, and influenza A virus (IAV), influenza B virus (IBV), respiratory syncytial virus (RSV), adenovirus (ADV), and *Mycoplasma pneumonia* (MP) results, were collected from electronic medical records. SPSS (version 21.0) and GraphPad Prism 9 software were used for statistical analysis and figure creation.

**Results:**

79,746 children tested positive for IAV IgM, and 3196 children tested positive for IBV IgM, with an overall positive rate of 28.5 % for IAV and 1.1 % for IBV. IAV infections peaked at 21,502 cases in March 2023. 80,699 children underwent RSV IgM testing from April to October 2023, with 5726 (7.1 %) testing positive. The apex of RSV infections occurred in May 2023, with 2140 cases. Regarding ADV, 100,460 children underwent testing from April to October 2023, with 1981 (11.9 %) testing positive. The pinnacle of ADV infections reached 4546 cases in November 2023. Concerning MP, 474,913 children underwent MP testing, with 73,833 (15.5 %) testing positive. The zenith of MP infections occurred in November 2023, with 25,291 cases. Further analysis revealed that the outbreaks of these pathogens are occurring earlier than in previous years. Additionally, our data showed that children aged >3 years accounted for 79.6 %, 87.8 %, 88.6 %, and 77.8 % of the total IAV‐positive, IBV‐positive, ADV-positive, and MP-positive children, respectively. Conversely, RSV primarily infected children <6 years.

**Conclusion:**

Various respiratory pathogens showed an epidemic trend in children among children post-COVID-19. These results indicated that we should pay timely attention to the epidemiological trends and characteristics of respiratory pathogens in children after the COVID-19 pandemic and provide relevant information for society and clinical practice.

## 1 Introduction

Compared with adults, children are more susceptible to human respiratory pathogens infection due to the incomplete maturation of the immune system [[1], [2], [3]]. Human respiratory viruses are the leading cause of diseases in humans, which can lead to a series of respiratory symptoms and disease severity, with high incidence rate and mortality rates worldwide, especially in children [4]. Meanwhile, *Mycoplasma pneumoniae* (MP) is a bacterium that can cause illness by damaging the lining of the respiratory system [5]. *MP* infection is one of the most common causes of community-acquired pneumonia (CAP) in children. Up to 10 % of *MP*-infected children developed pneumonia [3]. During the Coronavirus disease 2019 (COVID-19) period, the Chinese government implemented strict interventions, including wearing masks, maintaining social distancing, limiting crowd gatherings, and restricting outdoor activities to prevent the spread of COVID-19 [6]. Benefiting from the intervention measures for COVID-19, the incidence rate of human respiratory pathogens infection in children significantly decreased [7]. However, as emphasized by Messacar K et al. [8], the aftermath of the COVID-19 pandemic has resulted in a decline in active virus cases. Limited exposure to the virus and a shortage of vaccines have led to an increased susceptibility among the population.

With the announcement of the end of the COVID-19 pandemic, the children's lives, education, and social engagements have gradually returned to normalcy. This includes the cessation of mask-wearing mandates, the relaxation of social distancing requirements, and the resumption of in-person teaching. Consequently, there is a rising risk of outbreaks involving respiratory viruses and other pathogens associated with respiratory illnesses in children. From January 2023 to December 2023, children in China have encountered infections with various pathogens, including influenza A virus (IAV), respiratory syncytial virus (RSV), adenovirus (ADV), MP, and influenza B virus (IBV). Before the COVID-19 pandemic, the trend of various viral infections has a certain regularity, such as in Henan Province, China, influenza virus and ADV being more prevalent in (December to February of the following year) and spring (March to May), and RSV is more prevalent in winter [9]. However, information regarding the incidence rates, epidemic trends, and characteristics of these viruses and MP infections in children, as well as whether these epidemiological characteristics have changed, remains unclear. To address this gap, a single‐center retrospective observational study was conducted to assess the epidemiological characteristics of the mentioned pathogens in children in Henan, China.

## 2 Methods

### 2.1 Patient selection

A single‐center retrospective observational study was conducted at Henan Children's Hospital (Children's Hospital Affiliated to Zhengzhou University). From January 2023 to December 2023, a total of 575,373 children with respiratory symptoms such as fever and cough came to our hospital for relevant respiratory pathogen antigen/antibody testing were included in this study. The inclusion criteria for this study were children younger than 18 years of age. Among them, 279,976 children underwent influenza A and B antigen testing, 80,699 children underwent RSV antigen testing, 100,460 children underwent ADV antigen testing, and 474,913 people underwent antibody testing for MP. The study protocol complied with the Declaration of Helsinki and was approved by the hospital's ethics review board. The study protocol complied with the Declaration of Helsinki and was approved by the Ethics Committee of Henan Children's Hospital. Every research technique used in this study was considered part of regular clinical practice, and the information was kept anonymous. Given the study's retrospective nature, the informed consent requirement was waived.

### 2.2 Data collection and human respiratory pathogens measurements

The demographic and laboratory data, including age, gender, testing time, and IAV, IBV, ADV, and MP results, were obtained from electronic medical records. The Abbott Clearview influenza A and B test kit (Hangzhou Abo Biomedical Co., Ltd, China) was used to detect IAV and IBV antigens. The ADV and RSV antigen detection kit (Hangzhou Genesis Biodetection & Biocontrol Ltd, China) was used to detect ADV and RSV antigens. The MP IgM antibody detection kit (Bioneovan Co., Ltd. China) was used for MP IgM antibody detection. All the tests were performed according to the manufacturer's instructions.

### 2.3 Statistical analysis

All data analyses were performed using SPSS (version 21.0) software. Categorical variables were presented as frequencies and percentages. The figures were created with GraphPad Prism 9 software (GraphPad Software Inc.).

## 3 Results

### 3.1 Patient characteristics

This study enrolled 575,373 children who visited Henan Children's Hospital for IAV, IBV, ADV, RSV antigen, and MP-IgM antibody testing (Table 1). Among them, 279,976 children [154,543 boys (55.2 %) and 125,433 girls (44.8 %)] underwent IAV and IBV antigen testing, of which 15,879 (5.7 %) children were under 1 year old, 41,201 (14.7 %) children were between 1 and 2 years old, 98,240 (35.1 %) children were between 3 and 5 years old, and 124,656 (44.2 %) children were more than 6 years old. 80,699 children [45,433 boys (56.3 %) and 57,333 girls (43.7 %)] underwent RSV antigen testing, of which 11,511 (14.2 %) children were under 1 year old, 19,432 (24.1 %) children were between 1 and 2 years old, 28,630 (35.5 %) children were between 3 and 5 years old, and 21,126 (26.2 %) children were more than 6 years old. 100,460 children [56,157 boys (55.9 %) and 44,303 girls (44.1 %)] underwent ADV antigen testing, of which 8039 (8.0 %) children were under 1 year old, 17,868 (17.8 %) children were between 1 and 2 years old, 34,697 (34.5 %) children were between 3 and 5 years old, and 39,856 (39.7 %) children were more than 6 years old. 474,913 children [266,901 boys (56.2 %) and 208,012 girls (43.8 %)] underwent MP antibody testing, of which 38,865 (8.2 %) children were under 1 year old, 92,510 (19.5 %) children were between 1 and 2 years old, 186,096 (39.2 %) children were between 3 and 5 years old, and 157,442 (33.1 %) children were more than 6 years old. As mentioned above, there are significant differences in the number of children in different age groups who undergo virus and MP testing. Meanwhile, a total of 79,746 (28.5 %) children tested positive for IAV, 3196 (1.1 %) children tested positive for IBV, 5726 (7.1 %) children tested positive for RSV, 11,981 (11.9 %) children tested positive for ADV, and 73,833 (15.5 %) children tested positive for MP. Further analysis indicates significant differences in infection rates among different viruses and MP, with IAV having the highest infection rate, followed by MP, ADV, RSV, and IBV (Table 1).

**Table 1:** Characteristics of study participants.

**Variables**	**IAV**	**IBV**	RSV*	ADV*	**MP**	*χ*^*2*^	*P*
**Total number of visits**	279,976	279,976	80,699	100,460	474,913		
**Gender**						118.323	<0.001
**Male, n (%)**	154,543 (55.2 %)^a^	154,543 (55.2 %)^a^	45,433 (56.3 %)^b^	56,157 (55.9 %)^b^	266,901 (56.2 %)^b^		
**Female, n (%)**	125,433 (44.8 %)^a^	125,433 (44.8 %)^a^	35,266 (43.7 %)^b^	44,303 (44.1 %)^b^	208,012 (43.8 %)^b^		
**Age**							
< **1 years, n (%)**	15,879 (5.7 %)^a^	15,879 (5.7 %)^a^	11,511 (14.2 %)^b^	8039 (8.0 %)^c^	38,865 (8.2 %)^c^	8452.158	<0.001
**′1–2 years, n (%)**	41,201 (14.7 %)^a^	41,201 (14.7 %)^a^	19,432 (24.1 %)^b^	17,868 (17.8 %)^c^	92,510 (19.5 %)^d^	6733.274	<0.001
**3–5 years, n (%)**	98,240 (35.1 %)^a^	98,240 (35.1 %)^a^	28,630 (35.5 %)^a^	34,697 (34.5 %)^b^	186,096 (39.2 %)^c^	2142.748	<0.001
> **6 years, n (%)**	124,656 (44.2 %)^a^	124,656 (44.2 %)^a^	21,126 (26.2 %)^b^	39,856 (39.7 %)^c^	157,442 (33.1 %)^d^	19557.341	<0.001
**Positive, n (%)**	79,746 (28.5 %)^a^	3196 (1.1 %)^b^	5726 (7.1 %)^c^	11,981 (11.9 %)^d^	73,833 (15.5 %)^e^	89739.029	<0.001

Abbreviations: IAV, Influenza A virus; RSV, Respiratory syncytial virus; ADV, Adenovirus; MP, *Mycoplasma pneumoniae*.

Superscript letter: The same superscript letters between the two groups indicate no significant difference, and the different superscript letters between the two groups indicate significant difference.

* Note: Data collected from April 25th to December 31st, 2023.

### 3.2 Epidemiological trends of IAV, IBV, ADV, RSV, and MP infections in children

**Fig. 1:**
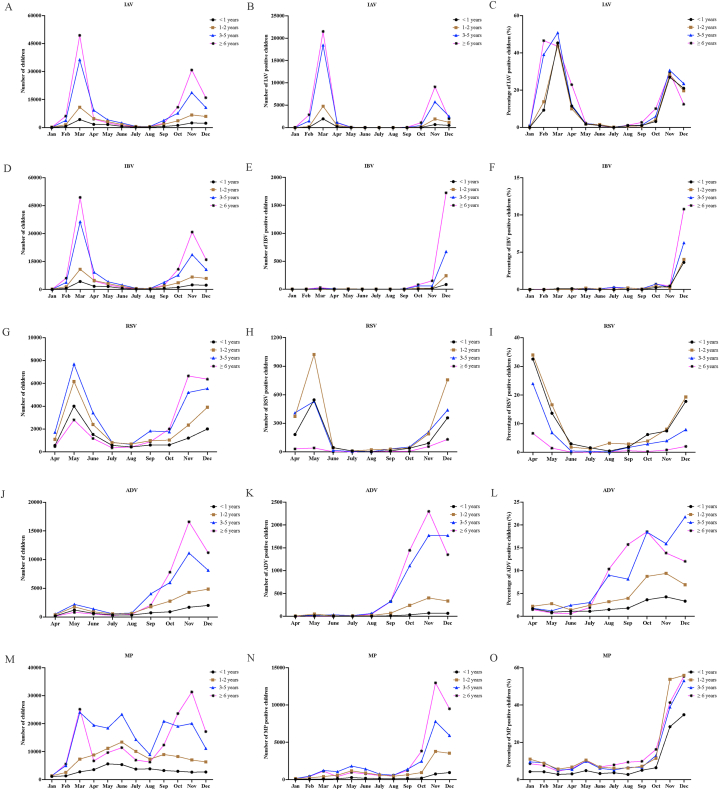
Epidemiological overview of IAV (Panel A–C), IBV (Panel D–F), RSV (Panel G–I), ADV (Panel J–L), and MP (Panel M − O) infections in children.

In order to evaluate the epidemiology of IAV, IBV, ADV, RSV, and MP infections in children, we counted the number of children who underwent the above testing items, the number of positive tests, and the positivity rate monthly. As shown in Fig. 1A, B, and 1C, IAV infections peaked at 21,502 cases (46.2 %) in March 2023, followed by a gradual decline in infected individuals and positivity rates. However, there has been a resurgence in the trend of IAV infections starting from October 2023, and the IAV infection reached a small peak in November 2023. From January to November 2023, the IBV showed a low prevalence trend, but in December 2023, the B influenza virus showed an epidemic trend (Fig. 1D, E, and 1F). Regarding RSV, the apex of RSV infections occurred in May 2023, with 2140 cases, after which the number and positive rate of RSV infections progressively decreased. However, starting in November 2023, RSV infections’ number and positivity rate increased (Fig. 1G, H, and 1I). Concerning ADV, starting from September 2023, the number of ADV infections began to rise and reached its peak in November 2023, with 4546 cases (Fig. 1J, K, and 1L). Currently, ADV infections are still ongoing. In October 2023, MP infections showed an epidemic trend in children. The zenith of MP infections occurred in November 2023, with 25,291 cases. Children with MP infection still have a high prevalence trend (Fig. 1M, N, and 1O).

### 3.3 Comparison of IAV-positive rates in children of different age groups

**Fig. 2:**
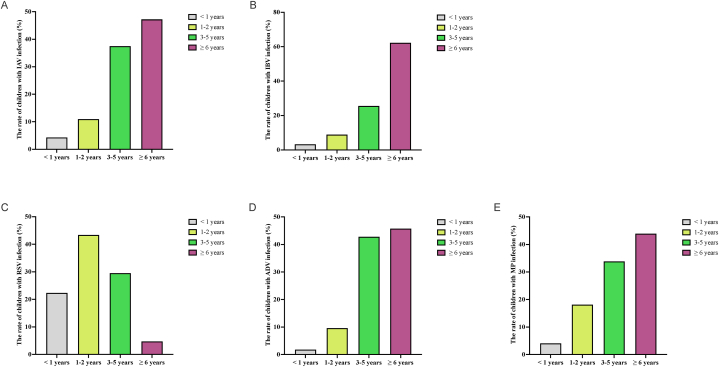
Number and percentage of children with IAV (A), IBV (B), RSV (C), ADV (D), and MP (E) infections across various age groups.

To further examine the epidemiological characteristics of the pathogens mentioned above in children across various age groups, we categorized them into four distinct age groups: <1, 1–3, 3–6, and >6 years. As depicted in Table 2, the breakdown of positive cases for IAV revealed 3423 (21.6 %), 8754 (21.1 %), 29,904 (30.4 %), and 37,665 (30.2 %) children within these age brackets, respectively. These figures corresponded to 4.3 %, 11.0 %, 37.5 %, and 47.2 % of children with IAV infection (Fig. 2A). Regarding IBV, 104 (0.6 %) infected patients were reported in the < 1-year-old group, 285 (0.7 %) in the 1–2‐year group, 816 (0.8 %) in the 3–5‐year group, and 1991 (1.6 %) in the >6‐year group (Table 2). These figures represented 3.3 %, 8.9 %, 25.5 %, and 62.3 % of children with RSV infection (Fig. 2B). In the case of RSV, 1280 (11.1 %) infected patients were reported in the < 1-year-old group, 2485 (12.8 %) in the 1–2‐year group, 1690 (5.9 %) in the 3–5‐year group, and 271 (1.3 %) in the >6‐year group (Table 2), which accounted for 22.4 %, 43.4 %, 29.5 %, and 4.7 % of children with RSV infection (Fig. 2C). For ADV, 215 (2.6 %) cases were observed in the < 1-year-old group, 1154 (6.1 %) in the 1–2‐year group, 5130 (12.9 %) in the 3–5‐year group, and 5482 (12.1 %) children in the >6‐year group (Table 2), which accounted for 1.8 %, 9.6 %, 42.8 %, and 45.8 % of children with ADV infection (Fig. 2D), respectively. Concerning MP infection in children, 3025 (7.8 %) cases were noted in the <1‐year group, 13,389 (14.5 %) in the 1–2‐year group, 24,992 (13.4 %) children in the 3–5‐year group and 32,427 (20.6 %) children in the >6‐year group (Table 2), which represented 4.1 %, 18.1 %, 33.8 %, and 43.9 % of children with MP infection (Fig. 2E), respectively. Further analysis shows significant differences in the positive rates of respiratory virus and MP infections among different age groups, with IAV being the highest and IBV being the lowest for children under <1 year old, 1–2 years old, and 3–5 years old. Regarding children aged >6 years, the IAV infection rate is highest, followed by MP, ADV, IBV, and RSV infection rates (Table 2).

**Table 2:** Number and percentage of children with IAV, IBV, RSV, ADV, and MP infections across various age groups.

**Variables**	**IAV**	**IBV**	RSV*	ADV*	**MP**	*χ*^*2*^	*P*
**Age groups**							
< **1 years, n (%)**	3423 (21.6 %)^a^	104 (0.6 %)^b^	1280 (11.1 %)^c^	215 (2.6 %)^d^	3025 (7.8 %)^e^	4970.584	<0.001
**1–2 years, n (%)**	8754 (21.2 %)^a^	285 (0.7 %)^b^	2485 (12.8 %)^c^	1154 (6.1 %)^d^	13,389 (14.5 %)^e^	9189.337	<0.001
**3–5 years, n (%)**	29,904 (30.4 %)^a^	816 (0.8 %)^b^	1690 (5.9 %)^c^	5130 (12.9 %)^d^	24,992 (13.4 %)^e^	37776.765	<0.001
> **6 years, n (%)**	37,665 (30.2 %)^a^	1991 (1.6 %)^b^	271 (1.3 %)^c^	5482 (12.1 %)^d^	32,427 (20.6 %)^e^	42502.511	<0.001

Abbreviations: IAV, Influenza A virus; RSV, Respiratory syncytial virus; ADV, Adenovirus; MP, *Mycoplasma pneumoniae*.

Superscript letter: The same superscript letters between the two groups indicate no significant difference, and the different superscript letters between the two groups indicate significant difference.

* Note: Data collected from April 25th to December 31st, 2023.

## 4 Discussion

COVID-19 is a highly infectious disease caused by severe acute respiratory syndrome coronavirus 2, mostly resulting in life-threatening complications [10]. To prevent the spread of COVID-19, many countries implemented strict interventions, including wearing masks, maintaining social distancing, limiting crowd gatherings, and restricting outdoor activities [11]. These control measures taken during the COVID-19 pandemic had an impact on the spread and activity of the influenza virus and respiratory syncytial virus [12,13]. However, with the Chinese government's management of COVID-19 Category B on January 8, 2023, the strict intervention measures taken during the COVID-19 period were gradually relaxed. Subsequently, people gradually increased their social activities. Offline teaching was restarted in kindergartens and schools; thus, outdoor activities and offline schooling potentially increased the transmission of other respiratory pathogens.

Children are more susceptible to influenza illness and are more prone to further development into severe illness than adults due to the incomplete development and maturation of their immune system [1]. Meanwhile, in 2020, the population of Henan, a populous province, was 114.44 million, including 26.48 million children aged less than 14 years, accounting for 23 % of the total population. With the lifting of strict control measures for COVID-19, many children are under the threat of respiratory pathogens outbreak. Therefore, real-time dynamic detection of respiratory pathogen infections in children and the characteristics of the infected population is helpful for early intervention and prevention and has important clinical significance in preventing the further spread of respiratory disease epidemics.

In this study, distinct trends were observed in the occurrence of respiratory viruses following the resolution of COVID-19. Specifically, the IAV outbreak manifested in March 2023, while the RSV epidemic emerged in May 2023. Subsequently, the outbreaks of ADV and MP commenced in October 2023. Contrasting these trends with the epidemiological patterns of related pathogens pre-COVID-19, we noted that influenza B exhibited an epidemic surge from 2021 to 2022 during the active COVID-19 period [14]. For IAV, Wang et al. [7], reported that The positivity for influenza A virus decreased from 22.5 % in 2019 to 9.9 % in 2020 to 0.2 % in 2021, which was attributed to the strong “intervention” in response to the COVID-19 pandemic. After the COVID-19 pandemic, IAV experienced an outbreak in March 2023, with an infection rate peaking at 46.2 %. Monitoring data indicates that IAV has sustained an epidemic trend since October 2023, reaching a small peak in November 2023. Comparing the positive rate of influenza A before, during, and after the COVID-19 pandemic, we found that the positive rate of IAV after the pandemic was higher than that before and during the COVID-19 pandemic. Before the advent of COVID-19, RSV demonstrated seasonal fluctuations annually between September and May from 2017 to 2019 [15]. However, in 2023, the RSV outbreak commenced in April and reached its zenith in May, with an infection rate of 26.0 %, which is higher than the RSV infection rate before the COVID-19 pandemic in Shanghai and Hangzhou, China [16,17]. In contrast to the typical prevalence of ADV during winter [18], our data revealed an ADV epidemic in September 2023, with monitoring data indicating its persistent prevalence. The ADV infection rate among children in Henan exceeded the previously reported rates (4.04 %) in children from 2009 to 2020 [19]. Unlike the usual prevalence of MP in northern China during summer and autumn, the prevalence trend of MP in the 2023 post-COVID-19 pandemic was in October, with an overall positive rate of 15.5 %. This rate was lower than the MP positive rate (13.08 %) reported in Beijing from 2016 to 2020 and higher than in 2021 [20]. Furthermore, our data underscored that children aged >3 years accounted for 79.6 %, 87.8 %, 88.6 %, and 77.8 % of the total IAV‐positive, IBV‐positive, ADV-positive, and MP-positive children, respectively. Conversely, RSV primarily infected children <6 years, with the age group representing 95.3 % of the total positive population. More importantly, compared to the research of H Yang et al., our data showed that the number of children infected with IAV, IBV, ADV, and RSV increased in the 2023 post-COVID-19 pandemic. The increased number of children infected with various viruses may be due to reduced exposure to external pathogenic microorganisms and vaccine shortages, resulting in immune debt [19].

Nonetheless, our study has some limitations. First, the respiratory pathogens antigen/antibody was tested using the rapid colloidal gold method, which might have led to some false positives or negatives. Second, due to the start of AVD and RSV antigen testing on April 25, 2023, data on ADV and RSV antigen testing from January to April 25, 2023, is missing from this study. Thirdly, as our hospital has not conducted any other respiratory viruses-related tests for children, this study was unable to display the data related to other viral infections. Lastly, this was a retrospective, single-center, cross-sectional observational study that requires further validation from multiple centers.

## 5 Conclusion

In summary, various respiratory pathogens have surfaced following the end of the COVID-19 pandemic and the relaxation of epidemic prevention and control measures, exhibiting infection rates surpassing those observed during the COVID-19 pandemic. Meanwhile, the outbreaks of these pathogens are occurring earlier than in previous years, warranting our attention to provide timely epidemiological trends of related pathogens for society and clinical practice. At the same time, in the post-COVID-19 pandemic, adopting appropriate non-pharmaceutical personal protection measures is imperative. These measures include wearing masks, regular hand sanitization, and maintaining social distance, as they prove instrumental in preventing infections caused by various respiratory pathogens.

## Ethics approval

The study was conducted according to the principles of the Declaration of Helsinki and was approved by the Hospital Ethics Review Board of Henan Children's Hospital, Zhengzhou, China (2024-K122). The study guarantees that the identity of the participants and other related data has been kept anonymous and confidential. The requirement for obtaining informed consent was waived owing to the study's retrospective nature.

## Funding

This work was funded by the 10.13039/501100001809National Natural Science Foundation of China (82200097), Key Research, Development, and Promotion Projects of Henan Province (232102310235 and 232102310122), and the Medical Science and Technology Project of Henan Province (LHGJ20220774).

## Data availability statement

Data will be made available on request.

## CRediT authorship contribution statement

**Tiewei Li:** Writing – original draft, Funding acquisition, Data curation, Conceptualization. **Xiaojuan Li:** Visualization, Methodology, Formal analysis, Data curation. **Xinrui Liu:** Writing – review & editing, Supervision, Data curation, Conceptualization. **Lili Dong:** Data curation. **Hui Fu:** Visualization, Formal analysis, Data curation. **Fatao Lin:** Visualization, Formal analysis, Data curation. **Yu Tang:** Visualization, Formal analysis, Data curation. **Zhipeng Jin:** Writing – review & editing, Writing – original draft, Conceptualization.

## Declaration of competing interest

The authors declare that they have no known competing financial interests or personal relationships that could have appeared to influence the work reported in this paper.
